# COVID‐19 vaccination‐linked granuloma annulare in two patients

**DOI:** 10.1002/ski2.412

**Published:** 2024-07-24

**Authors:** Emma McIntyre, Philina Lamb, Maxwell A. Fung, Maija Kiuru, Lawrence S. Chan

**Affiliations:** ^1^ Department of Dermatology UC‐Davis School of Medicine Sacramento California USA

## Abstract

The COVID‐19 pandemic brought not only a huge healthcare challenge to the world but also introduced many questions of how the human immune system reacts to counter viral invasion, including vaccination. Unlike most vaccinations that are not usually linked to any specific skin eruptions, COVID‐19 vaccination has been linked to a variety of skin lesions. In this paper, we present two patients who developed granulomatous skin lesions post‐COVID‐19 vaccination, one patient has generalised granuloma annulare (GA) and the other patient develops localised GA. Both patients have good responses to treatment regimens containing systemic corticosteroids. We review the literature pertaining to COVID‐19‐linked skin diseases, particularly granulomatous diseases and discuss the possible pathomechanism of granulomatous eruptions in relation to COVID‐19 vaccination.

## Graphical abstract

Generalised or localised granuloma annulare (GA) can be observed in patients who received RNA‐directed COVID‐19 vaccination. This granulomatous skin reaction seems to be responding to systemic corticosteroid. GA may be considered as an immunological reaction to RNA‐directed vaccination.

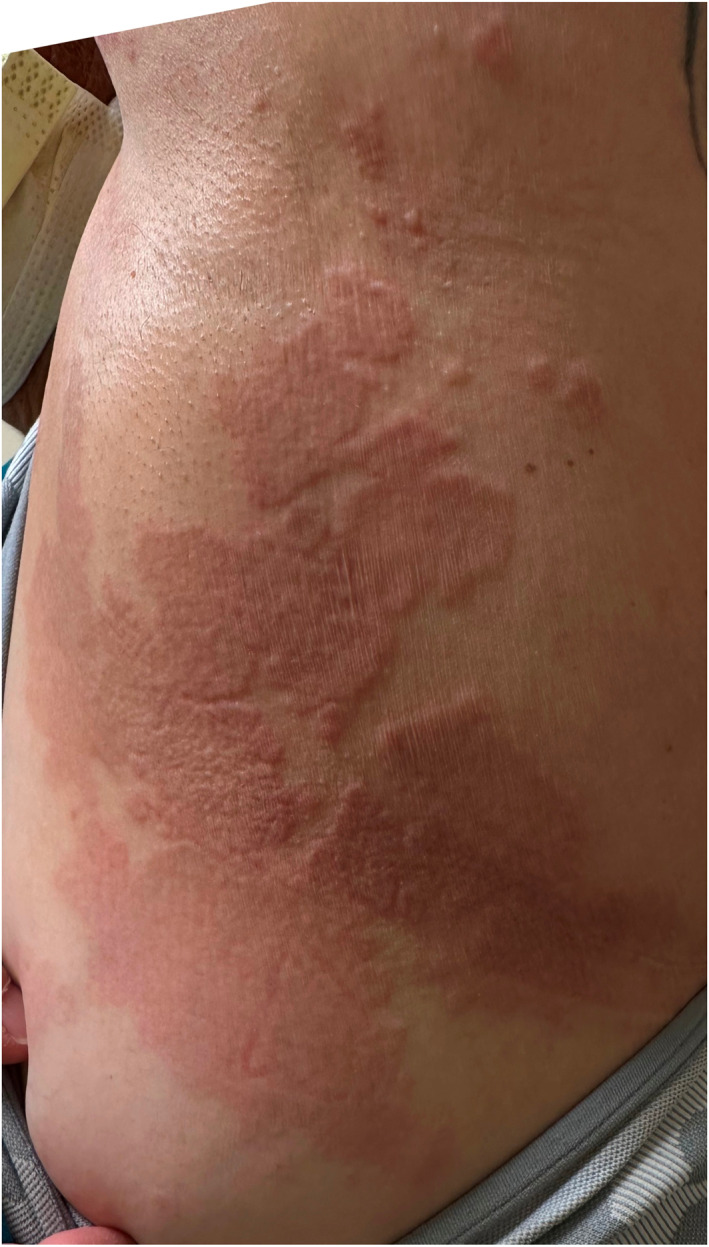

## 1 INTRODUCTION

COVID‐19 infection has been known to be associated with a variety of skin manifestations including semi‐defined eruptions, such as vaso‐occlusive lesions, vesicular lesions, morbilliform rash, urticarial lesions and pseudo‐chilblains. Defined eruptions include autoimmune‐mediated IgA vasculitis and IgA‐nephropathy.^1^, ^2^, ^3^ Dermatologists are familiar with skin findings in relationship to viral infections, such as pityriasis rosea (with human Herpesvirus‐6 and ‐7), erythema multiforme (with Herpes simplex virus) and porphyria cutanea tarda (with hepatitis C).^4^ Vaccinations against viral infections, on the contrary, are not commonly associated with skin manifestations, particularly a generalised rash. Vaccinations against COVID‐19 infection, nevertheless, have been linked to several skin rashes. These skin reactions include delayed injection site reaction, urticaria, morbilliform rash, erythromelalgia, vesicular lesions, chilblains, angioedema, pityriasis rosea, erythema multiforme, vasculitis, lupus erythematosus, immune thrombocytopaenia and petechiae.^5^, ^6^ Recently, the first case of generalised granuloma annulare (GA) has been linked to such vaccinations.^7^ We are reporting in this paper, a generalised GA and a localised GA following COVID‐19 vaccination that worsened after receiving subsequent vaccinations or boosters. We reported these cases to alert the dermatology community to the possible link between COVID‐19 vaccination and granulomatous skin manifestations. Furthermore, possible immunologic mechanisms behind such associated findings are discussed.

## 2 CASE REPORT

**FIGURE 1:**
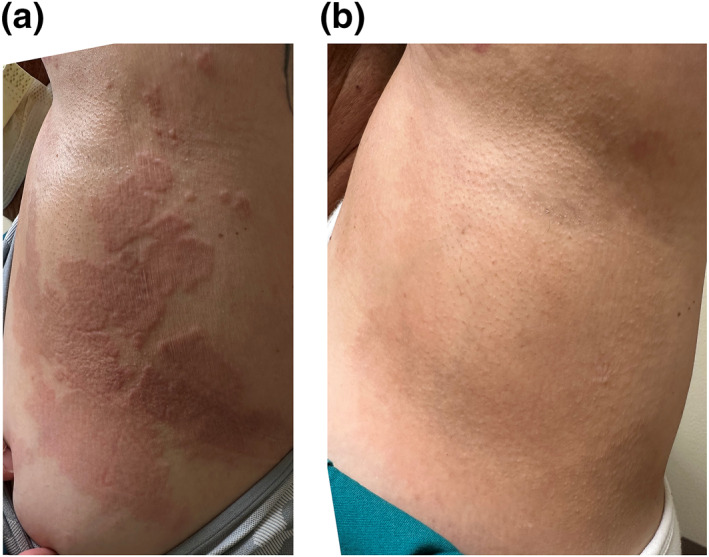
(a) Clinical manifestations of a generalised granuloma annulare in the left torso of a female patient receiving COVID‐19 vaccination (Case 1). (b) Significant clinical resolution observed in the left torso of patient 1 after treatment.

**FIGURE 2:**
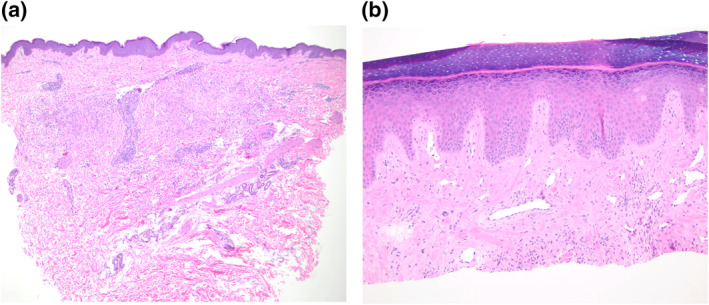
(a) Histopathology of a left flank skin lesion showing an interstitial and palisaded histiocytic infiltrate surrounding degenerated collagen in the dermis, consistent with granuloma annulare (Case 1). (b) Shave biopsy from the dorsal hand showed the upper portion of an interstitial histiocytic infiltrate compatible with interstitial GA (Case 2). Magnifications: 4X(a), 10X(b).

**FIGURE 3:**
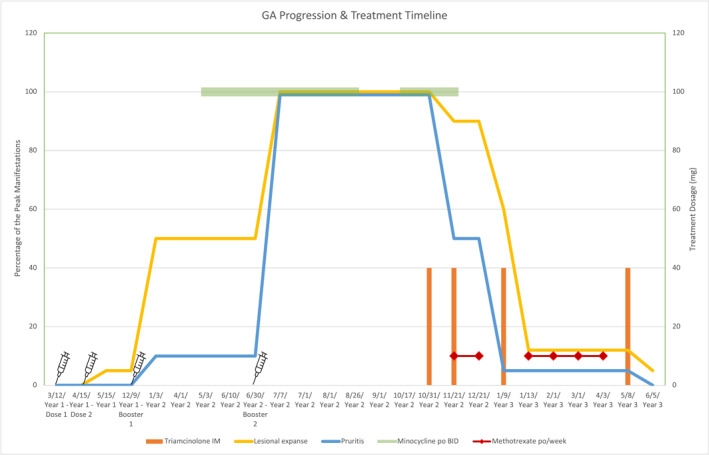
Temporal relationship documenting COVID‐19 vaccination, vaccine boosters and treatments and the occurrence and resolution of pruritus symptom and skin rash of patient case 1.

Case 1. A 54‐year‐old female patient presents to an academic dermatology department for a granulomatous eruption that involves her thigh, abdomen, torso, and groin areas, covering 25% of her total body skin surfaces (Figure 1a). Initially, three small red papules over her lower abdomen surfaced about one month after receiving the second dose of Pfizer's COVID‐19 vaccine. Later, a more generalised rash of similar morphology developed about one month after her first booster of COVID‐19 vaccine. Subsequently substantial worsening of her rash 1 week after the second booster of the same vaccine occurred. The rash was not symptomatic at the onset. However, pruritus was noticeable after the first booster and became more intense after the second booster. A skin biopsy performed after the first booster revealed the following histological findings: a multifocal, palisaded and interstitial histiocytic infiltrate with central degenerated collagen in the dermis, consistent with a diagnosis of GA (Figure 2a). The patient initially received minocycline 100 mg twice daily, one‐time intralesional triamcinolone injection (5 mg/cc, total 1.5 cc) and triamcinolone cream. This regimen helped minimally, and thus was discontinued. When the patient's rash got worse after the second booster, minocycline was restarted. Two weeks later, 40 mg intramuscular triamcinolone was given to the patient, resulting in 50% of reduction in pruritus and 10% reduction in redness of the lesions. Thus, additional 40 mg intramuscular triamcinolone was again administered. Minocycline was discontinued (due to gait instability reported by the patient). In addition, methotrexate 10 mg/week was started and continued for 4 weeks. On a subsequent office visit, the patient's pruritus was reduced to 5% of the peak level, and 40% lesions were essentially flattened hyperpigmented patches without erythema. Methotrexate 10 mg per week was continued for the next 2.5 months without additional improvement and was discontinued. On the most recent office visit, all lesions were flattened, and most were without erythema (Figure 1b). The disease progressions and therapeutic responses are depicted in relationship to the timeline of vaccinations and treatments (Figure 3).

Case 2. A 66‐year‐old male patient presented to an academic dermatology department for a localised skin lesion. He had received the first dose of Pfizer's COVID‐19 vaccination a few weeks prior to the onset of lesion. The initial lesion, which was pruritic and non‐tender, surfaced on the interphalangeal joint of his right dorsal thumb as a 1‐cm well‐demarcated pink ovoid plaque. The lesions were subsequently spread to his left dorsal hand and knee in the next few weeks after receiving the second dose. A biopsy on the dorsal thumb revealed the upper portion of an interstitial mononuclear cell infiltrate with few multinucleated cells associated with thickened collagen fibres, compatible with a diagnosis of interstitial GA (Figure 2b). The patient received one dose of intralesional triamcinolone (10 mg/cc) and topical corticosteroid betamethasone for treatment, which improved his pruritic symptom. His left knee lesion was completely resolved, and his right thumb lesion was reduced to a 6‐mm‐size papule.

## 3 DISCUSSION

There are many predisposing factors in patients who develop vaccine hypersensitivity with the strongest association being previous allergic reaction to a vaccine or its components.^8^ Other risk factors for adverse drug reactions in general include older age, concomitant disease states, female sex and the ability of the drug to act as a hapten.^9^ It has also been hypothesised that repeated intermittent drug administration—such as the COVID‐19 vaccination schedule—is more sensitising than continuous drug treatment.^9^ However, unless the patient has a severe anaphylactic reaction to vaccination, it is recommended to continue vaccination as scheduled and if possible, identify the allergen and opt for an allergen‐free vaccine.^8^

**FIGURE 4:**
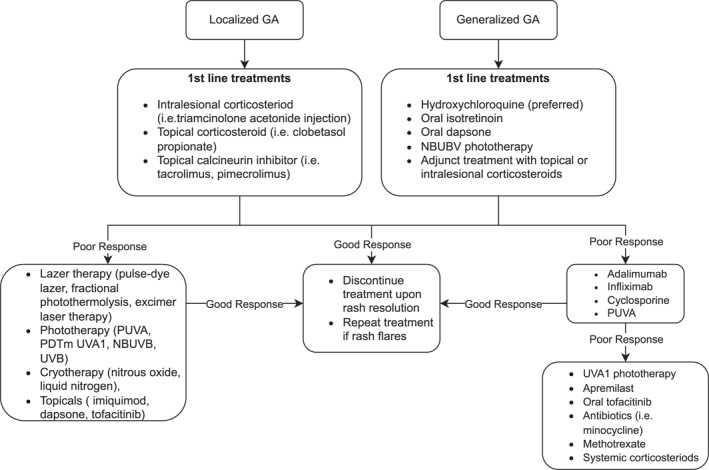
Algorithm for the treatment of granuloma annulare (GA).^13^

Diagnosis and classification of vaccine hypersensitivity can be challenging. If the reaction onset is within minutes to hours, one must distinguish between a true anaphylactic reaction and vasovagal syncope.^8^ If the reaction is a true anaphylactic reaction—characterised by hypotension, wheezing, tachycardia and hives—treatment with epinephrine is indicated.^8^ Symptoms of delayed type hypersensitivity to vaccination are often characterised by large local reactions and chronic subcutaneous nodules with pruritus.^8^ These symptoms, which were present in the two cases reported here, often resolve with antihistamine and topical or systemic corticosteroids.^10^ Topical and intralesional corticosteroids are typically first line treatment for both localised and generalised GA, whereas hydroxychloroquine is especially efficacious for generalised GA.^11^ Although hydroxychloroquine is typically the first line for generalised GA, in our first case patient, minocycline was initiated by an outside physician for unknown reasons. A review by Joshi and Duvic suggested that if first line treatment with corticosteroids fails, other treatment options to consider are methotrexate, phototherapy, immunomodulators—such as apremilast—or biologics—such as adalimumab.^12^ Figure 4 further outlines the differences in the management of generalised versus localised GA.^13^

Vaccine‐induced GA is exceedingly rare, but of the cases reported, most occurred after the *Bacillus* Calmette–Guerin (BCG) vaccine. A recent study by Garcia‐Gil et al. found 13 cases of GA following vaccination: eight being after BCG, two after Hepatitis B, and single cases after influenza, tetanus, diphtheria‐tetanus toxoid, and pneumococcal vaccines.^14^ Ten of the aforementioned cases resulted in generalised GA, with most being single‐dose vaccines, and GA often resolved with topical corticosteroids.^14^ However, there were two cases of subsequent vaccination—one with Hep B and one with tetanus vaccination—causing the recurrence of generalised GA.^14^ Additionally, several cases of GA have been reported after COVID‐19 infections. Monte‐Serrano et al. reported one such case and suggested that the occurrence of GA could be explained by the activation of the immune system in response to COVID‐19 rather than the virus directly.^15^ Since infection with COVID‐19 can trigger GA, it follows that COVID‐19 vaccination which stimulates the immune system in a comparable way to active infection could also trigger GA. Although most GA are idiopathic in nature, our cases show a strong temporal association with COVID‐19 vaccination.

It should be noted that the clinical manifestations of our first patient are strikingly similar to a case study reported by Nguyen et al.^7^ Our first patient and the one described by Nguyen et al. are similar in age and sex, onset of rash and pruritus following COVID‐19 vaccination and generalised distribution of the GA rash across the thighs, abdomen, torso and groin. Both cases had no reported reaction to their first COVID‐19 vaccine but began developing GA two weeks after their second dose of the vaccine. Soon after receiving their first COVID‐19 booster, both patients manifested with more generalised GA and more pruritus. Interestingly, all three patients, our two patients and the one reported by Nguyen et al., developed GA after receiving vaccines manufactured by Pfizer Pharmaceutical and improved after receiving treatment regimens containing systemic corticosteroid.

The vaccination timeline and rash flares suggest an immunological link between COVID‐19 vaccination and granuloma. Vaccines contain antigens—such as toxoids or a microbial component to elicit an immune response, as well as additives—such as egg, gelatin and preservatives.^16^ Usually, vaccine hypersensitivity is due to the additive component, but rarely it can be due to the microbial antigen itself.^16^ While local vaccine‐induced hypersensitivity reactions are common, it is rare for a vaccine to cause a delayed onset T‐cell mediated systemic reaction.^16^ Nevertheless, Nguyen et al. hypothesised a Type IV hypersensitivity reaction as an explanation for their case of COVID‐19 vaccination‐associated GA.^7^ Our patients' GA, especially the first case of generalised GA, could also be attributed to a Type IV hypersensitivity reaction. Ultimately the pathogenesis of GA is unknown, although many mechanisms have been proposed. Early studies based on GA biopsy findings of IgM, complement, and fibrinogen in blood vessels led to the belief that type three immune response leading to chronic vasculitis was the mechanism of action.^17^ More recently, however, it has been proposed that the aforementioned biopsy findings are secondary to a type‐four immune response in which the activation of macrophages and fibroblasts is the primary mechanism of pathogenesis of GA.^17^ Further support for the type‐four immune response hypothesis is that GA biopsy specimens have been found to have no immune‐complex deposition on direct immunofluorescence and that helper T‐cells make up a large portion of the GA infiltrate on biopsy.^17^

GA has been associated with a wide variety of health conditions, including diabetes, dyslipidemia, malignancy, HIV, other viral infections and skin trauma.^18^ In terms of skin trauma, there have been cases of GA occurring after tattoo, vaccine, insect bite, herpes zoster eruption and even sun exposure.^18^ It has been proposed that the cases of skin trauma‐associated GA be studied using the concept of immunocompromised cutaneous district (ICD), wherein damaged skin acquires immune dysregulation, thus making it susceptible to granulomatous disease.^18^ ICD has many proposed mechanisms of immune dysregulation, but the two most studied are retention of exogenous antigen and altered neural signalling.^18^ In the retention of exogenous antigen hypothesis, it is proposed that vaccines introduce foreign material into the skin and can theoretically trigger a foreign body granulomatous response, but this does not explain how most cases of vaccine‐induced GA are generalised, and often spare the vaccination site.^18^ Concerning the hypothesis of altered neural signalling, it has been proposed that vaccine‐induced GA could be partially explained by peripheral nerve damage causing peptidergic fibres to modulate the local neuroimmune environment to favour granuloma formation.^18^ While there is evidence of damage to peptidergic fibres in herpes zoster infection and surgical traumas/burns, it is unclear if minor skin trauma such as vaccination can cause neurally driven granulomatous reactions.^18^

Given that there are now three cases—our two patients and the patient documented by Nguyen et al—with onset of GA following anti‐viral vaccination, in addition to the reported cases of GA linked to COVID‐19 infection, it is possible that GA has an RNA virus aetiology.^15^ It is interesting to note that many of the infections typically associated with GA (i.e. EBV, herpesvirus and HIV) establish true latency in their host—something that RNA viruses such as SARS‐COV‐2 are not typically known to do, likely due to their inability to integrate into the eukaryote genome. It is possible that SARS‐COV‐2 infection mimics these chronic forms of viral infection (EBV, herpesvirus and HIV) that are associated with GA by means of immune evasion leading to more chronic immune dysregulation, and thus predisposing patients to develop GA.

## CONFLICT OF INTEREST STATEMENT

None to declare.

## AUTHOR CONTRIBUTIONS

**Emma McIntyre**: Formal analysis (equal); writing – original draft (equal); writing – review & editing (equal). **Philina Lamb**: Data curation (equal); writing – review & editing (equal). **Maxwell Fung**: Data curation (equal); writing – review & editing (equal). **Maija Kiuru**: Data curation (equal); writing – review & editing (equal). **Lawrence S Chan**: Conceptualisation (lead); data curation (equal); formal analysis (equal); writing – original draft (equal); writing – review & editing (equal).

## ETHICS STATEMENT

Not applicable.

## PATIENT CONSENT

Written patient consent for publication was obtained.

## Data Availability

All the data contained in this manuscript can be validated from UC Davis Health Medical Record System.
